# Decision-Making of Underwater Cooperative Confrontation Based on MODPSO

**DOI:** 10.3390/s19092211

**Published:** 2019-05-13

**Authors:** Na Wei, Mingyong Liu, Weibin Cheng

**Affiliations:** 1 School of Marine Science and Technology, Northwestern Polytechnical University, Xi’an 710072, China; 2 Shaanxi Key Laboratory of Measurement and Control Technology for Oil and Gas Well, Xi’an Shiyou University, Xi’an 710065, China; wbcheng@xsyu.edu.cn

**Keywords:** autonomous underwater vehicle, cooperative confrontation, target allocation, multi-objective discrete particle swarm optimization (MODPSO)

## Abstract

This paper proposes a multi-objective decision-making model for underwater countermeasures based on a multi-objective decision theory and solves it using the multi-objective discrete particle swarm optimization (MODPSO) algorithm. Existing decision-making models are based on fully allocated assignment without considering the weapon consumption and communication delay, which does not conform to the actual naval combat process. The minimum opponent residual threat probability and minimum own-weapon consumption are selected as two functions of the multi-objective decision-making model in this paper. Considering the impact of the communication delay, the multi-objective discrete particle swarm optimization (MODPSO) algorithm is proposed to obtain the optimal solution of the distribution scheme with different weapon consumptions. The algorithm adopts the natural number coding method, and the particle corresponds to the confrontation strategy. The simulation result shows that underwater communication delay impacts the decision-making selection. It verifies the effectiveness of the proposed model and the proposed multi-objective discrete particle swarm optimization algorithm.

## 1. Introduction

Autonomous underwater vehicles (AUVs) have been used increasingly in the civilian and military field with their mobility and concealed advantages. In the civil sphere, AUVs are used for data collection, laying pipelines, environmental exploring, and so on. In the military area, they are applied for investigation, detection, target attacking and so on. As a significant combat force, AUVs has been widely used in naval battles. The manifestation of naval war has changed from a single weapon confrontation to a formation confrontation as in the military model. Moreover, the AUVs formation confronts more than one threat targets. Therefore, it is especially important to scientifically and reasonably research the target allocation decision-making of underwater cooperative confrontation.

Target allocation is an essential factor in an underwater confrontation. It is a fundamental problem arising in defense-related applications of operations research. As a classic constrained optimization problem developed in the field of military operations research, the purpose is to find the best solution for distributing weapons to the targets of opponents and to maximize the overall expected effect. Target allocation is an inherently nonlinear combinatorial optimization and standard nondeterministic complete polynomial issue [1,2,3,4,5]. As a hot research area, it has received extensive attention from researchers in various countries. Hungarian algorithm [6,7], genetic algorithm [8,9], ant colony algorithm [10,11], particle swarm algorithm [12,13] and so on, have been applied to target allocation and have achieved many results. Adaptive chaos parallels the clonal selection algorithm [14] and combined the benefits of chaos theory with parallel population classification, in order to realize the population initialization and population update. It solved the weapon target allocation (WTA) of the warship formation antiaircraft application. [15] explored the different game methods of acoustic sensor node cooperation in underwater cooperation and he compared the interaction and performance between nodes under different measurements. [16] used safety margins to develop optimal allocation problems and introduce optimization practices, the proposed optimization method integrated evolutionary games and particle swarm optimization to improve optimality and reduce computational load. The variable neighborhood search (VNS) algorithm [17] and the large-scale neighborhood (VLSN) search algorithm [18] obtained almost optimal results to solve moderately large size instances of the air combat WTA optimally. However, as the number of targets increased, the computational complexity made the solution process very long and it was only able to solve small-scale problems online. Although these proposed algorithms for solving target allocation had achieved fruitful results, they all ignored the discrete features of decision making. Therefore, it is necessary to propose an algorithm that is available in cooperative confrontation decision-making. The particle swarm optimization (PSO) algorithm [19,20] provides a new idea for solving this kind of issue. It was proposed by an American psychologist, Kennedy, and an electrical engineer, Eberhart, in 1995. The PSO simulated the phenomenon of birds looking for food in nature and had the characteristics of concise concept, timely implementation, and fast convergence [21]. As the algorithm has the advantages of easy application and fast optimization, it is naturally applied to solving the multi-objective optimization problems.

There are two basic types of target allocations which are static WTA (SWTA) and dynamic WTA (DWTA). In SWTA, all weapons are used to strike targets at the same stage, and it is necessary to find the optimal weapon allocation for temporary defensive tasks. DWTA is a multistage problem, which needs to consider the entire defense process and find the optimal global allocation. Given the underwater cooperative countermeasure of AUVs, [22] studied the dynamic cooperative attack and defense strategy of multiple AUVs. The established WTA single-objective model only considered the optimization of damage performance. In [23], the target allocation model of multi-AUVs based on the dynamic game was established by considering the survival probability and underwater environmental impact, however, the survival probability was still the primary indicator. In [24], the two factors of maximizing operational efficiency and minimizing operating costs were considered as resource constraints, and the multi-objective optimization problem was transformed into a single-objective optimization problem by maximizing the cost-benefit ratio as an objective function. Most existing models are based on the highest damage probability (or the opponent’s lowest survival probability) where the only consideration is to increase the damage probability and thoroughly allocate the weapon at the target. These models do not meet the reality of modern combat weapon distribution. In [25], recognizing this problem and proposing improvements, a new constraint variable was added to the original objective function to construct a complex objective function, but it was still a single target optimization. In [26,27,28] the weapon target allocation model only considered the optimization of damage effectiveness or the value of the protected asset, regardless of operational consumption, and only modelled the WTA problem as a single-objective optimization problem. In actual combat, the appropriate WTA model must not only meet specific tactical requirements but also consider issues such as weapon consumption during an antagonistic process. Therefore, the target decision problem in the actual confrontation process is a combinatorial optimization problem with multivariate and multiple constraints. Considering two or more objective functions is more practical. For example, common objective functions include minimizing weapon consumption, maximizing damage to threat targets, minimizing combat time consumption, and so on. The dual-target WTA problem was studied by [29] where the objective was to minimize the target cumulative survival probability and minimize the accumulative cost of the weapon target allocation. On the basis of the information from studies by [29,30] which considered the influence of the decision-time window on the number of weapon systems reused, a three-objective WTA model was established.

In practice, the WTA problem has many strong constraints that are strictly related to the actual situation, such as weapon quantity, feasibility, and fire constraints. Reference [31] under the premise of resource, feasibility and fire constraints, while maximizing the damage to the threaten targets and minimizing the consumption of ammunition, [31] proposed the NSGA-II using an adaptive strategy and a multi-objective optimization algorithm based on adaptive decomposition as a solution. For a similar model [31,32] used MOEA/D with an adaptive weight adjustment to solve it. Although there have been many related studies on the issue of weapon-target allocation, under the strong underwater constraints, none of them are suitable for underwater cooperative confrontation. In a real underwater confrontation, the complexity of underwater operations, the marine environment, the accuracy of sensor detection, and the state of communication will all affect the outcome, thus affecting the decision-making results. Although the single objective function can add conditional constraints, the form is more complex and unintuitive, and it can only provide one decision-making option that is less adaptable to changes in the battlefield situation. With further study of the complexity of naval warfare, the influence of underwater constraints on strategic choices should be considered, and a multi-objective decision-making optimization model should be constructed to solve the underwater synergistic confrontation decision-making optimization.

This paper considers the influence of underwater communication delay and constructs a multi-objective optimization decision-making model with minimum residual threat probability and minimum weapon consumption as the objective function to solve the underwater cooperative confrontation decision-making problem. The discrete particle swarm optimization (PSO) algorithm is used to represent the particle position as a candidate strategy for weapon target allocation through natural number coding. The allocation strategies are combined with the coding and update of the algorithm. The communication delay has an impact on optimal underwater cooperative allocation strategies.

The rest of this paper is structured as follows: Section 2 introduces the underwater sonar signal processing method and mathematical description of underwater cooperative confrontation decision-making. Section 3 describes the multi-objective discrete particle swarm optimization (MODPSO) algorithm for solving underwater cooperative confrontation decision-making model. Next, Section 4 carries out the simulation experiments, followed by an analysis of the performance. Finally, Section 5 gives the conclusion.

## 2. Problem Formulation

Ensuring that the decision-making model is more adaptive to the battlefield environment, our goal is to reduce weapon consumption as much as possible and to consider the decision preferences of the commander. This section will focus on the sonar signal recovery, separation, and the mathematical description of underwater cooperative confrontation decision-making.

### 2.1. Underwater Sonar Signal Processing Method

The goal of accurate detection is the foundation of attacking the target precisely. Due to the particularity of the multi-AUV working environment, the complex sensor system composed of a variety of sensors has become an indispensable part of the AUV. At present, the sensors used by AUVs mainly include sensors for sonar, positioning, laser ranging, vision, infrared, inertia, and acoustic sensitivity. A sonar sensor uses the ultrasonic reflection principle to detect the external environment, obstacles, and other positional information. Therefore, the AUV relies on the sensor system to achieve dynamic real-time obstacle avoidance and formation coordination, and to perceive local news in a dynamic unknown marine environment.

The accuracy of underwater sonar detection directly affects the strategy adopted by the both sides. Due to the disturbance of the sea surface and the seabed reverberation, ocean noise and self-noise will reduce the performance of the sonar detection. In order to improve the correct rate of sonar detection, it is necessary to remove the additive noise in the sonar receiving array and separate the denoised mixed sources one by one in the process of detecting and optimizing the sonar signals.

#### 2.1.1. Acoustic Sensor Signal Recovery

Suppose the source with additive noise is:

(1) $$r(k)=r_{1}(k)+\ldots +r_{N}(k)+n(k)$$

The sonar array output signal of the underwater acoustic sensor is:

(2) $$s(k)=r(k)\times h(k)=(r_{1}(k)+\ldots +r_{N}(k)+n(k))\times h(k)$$

Recovery signal obtained by the channel equalizer is:

(3) $$y(k)=x(k)\times e(k)=(r_{1}(k)+\ldots +r_{N}(k)+n(k))\times h(k)\times e(k)$$

The equalizer e used to supplement the channel, and the actual channel influence h satisfy the Equation (4)

(4) $$h(k)\times e(k)=\sum_{i}h(i)e(k-i)\approx c\delta (k-k_{0})$$

where c is the constant coefficient for the source signal recover.

#### 2.1.2. Signal Separation

According to the principle of acoustic sensor signal recovery, it assumes that there are M sensors of the sonar receiving array, as shown in Equation (5).

(5) $$CUM_{4}(r_{i}(k)=E\left\{{\left|r_{i}(k)\right|}^{4}\right\}-2E^{2}\left\{{\left|r_{i}(k)\right|}^{2}\right\}-{\left|E\left\{r_{i}^{2}(k)\right\}\right|}^{2}$$

where $r_{i}(k)(i=1,2,\ldots ,N)$ is not a zero signal. It is based on N narrow-band, non-Gaussian, mutually statistically independent and fourth-order cumulant.

(6) $$y(k)=r(k)=r_{1}(k)+\ldots +r_{N}(k)+n(k)$$

where $n(k)={\left[n_{1}(k),n_{2}(k),\ldots ,n_{N}(k)\right]}^{T}n_{i}(k)(i=1,2,\ldots M)$ is the additive white Gaussian noise of the *i*^th^ array elements. The mean value is zero, the variance is $\delta^{2}$. The cost function based on the logarithm kurtosis maximization criterion is:

(7) $$y(\omega )=\frac{1}{4}\ln(\frac{\left|CUM_{4}[y(k)]\right|}{E^{2}[\left|y{\left(k\right)}^{2}\right|]})$$

The second signal source can be obtained by removing the first signal from the original mixed-signal, and the calculation formula is:

(8) $$y_{2}(k)=y_{1}(k)-{\hat{\omega }}_{2}d_{1(k)}$$

where $d_{1}(k)$ is the first separated signal source, ${\hat{\omega }}_{2}$ is the weight coefficient of the filter, which is an N-dimensional column vector.

(9) $$l_{1}({\hat{\omega }}_{1})=\frac{1}{p}{\left\|y_{2}(k)\right\|}^{p}$$

p is a positive integer between 4 and 8. Minimize $l_{1}({\hat{\omega }}_{1})$, then the iterative formula of ${\hat{\omega }}_{1}$ can be written as Equation (9).

(10) $${\hat{\omega }}_{1}(k+1)={\hat{\omega }}_{1}(k)+\eta_{1}(k)y_{1}(k)(s_{2}(\hat{k}+1))p-1$$

The recovered signal can be separated according to Equation (10).

### 2.2. Mathematical Description of Underwater Cooperative Confrontation Decision-Making

**Figure 1:**
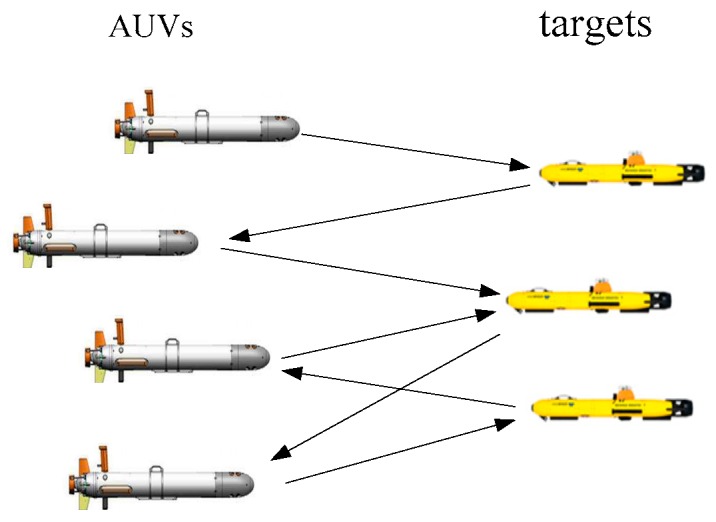
Allocation in the process of underwater cooperative confrontation.

In the process of underwater cooperative confrontation, AUVs need to attack or defend against one threat target or multiple threat targets. Therefore, target allocation is vital to solving the confrontation decision-making problem. Target allocation involves allocating various targets to system members in an operational process according to specific requirements. A reasonable target allocation strategy can improve the overall survival probability and operational effectiveness of AUVs. Figure 1 shows the target allocation in the collaborative confrontation process.

According to the form of confrontation between the two parties, target allocation can be divided into direct confrontation target allocation and indirect confrontation target allocation. Direct confrontation means that both the system members and the assigned targets can attack. The purpose of both sides is to destroy the other side directly. Therefore, in the process of target allocation, it is necessary to ensure the rational allocation of resources and to ensure that the threat of targets to system members is as small as possible. Indirect confrontation refers to an assigned target that has no attack power, and it can only evade attacks in a certain way. Therefore, the target allocation process needs to consider how to allocate resources reasonably so that the success rate of confrontation is the highest. This paper mainly addresses research on the indirect confrontational target allocation.

In the process of cooperative confrontation, the cost of attacking or defending the target is generally different, due to the difference in target “priority” and relative motion information. Therefore, the key to solving underwater target allocation is to describe the problem adequately and determine the corresponding allocation rules. In the collaborative attack problem, in order to not miss the real target, it is necessary to ensure that at least one AUV attacks each target. The decision variable $d_{ij}$ is defined to indicate the distribution relationship between the AUV *i* and the target *j*. $d_{ij}=1$ represents that AUV *i* is assigned to attack target *j*, otherwise $d_{ij}=0$. Target allocation strategies can be represented by the decision matrix (suppose there are $N_{t}$ targets and $N_{m}$ AUVs).

$$D=\left[\begin{array}{cccc} d_{11} & d_{12} & \ldots & d_{1N_{t}}\\ d_{21} & d_{22} & \ldots & d_{2N_{t}}\\ \vdots & \vdots & \ddots & \vdots \\ d_{N_{m1}} & d_{N_{m2}} & \ldots & d_{N_{m}N_{t}} \end{array}\right]$$

The constraints for underwater target allocation are described as following:

(11) $$\sum_{i=1}^{N_{m}}d_{ij}\geq 1\begin{array}{cc} & i\in \left\{1,2,\ldots ,N_{m}\right\} \end{array}$$

(12) $$\sum_{j=1}^{N_{t}}d_{ij}\geq 1\begin{array}{cc} & j\in \left\{1,2,\ldots ,N_{t}\right\} \end{array}$$

There four allocation scenarios that may be in the target allocation scheme are: a single AUV attacks a single target; a single AUV attacks multiple targets; multiple AUVs collaboratively attack a single target; and multiple AUVs collaboratively attack multiple targets. This paper studies the fourth scenario.

In multiple AUVs cooperative naval warfare, the purpose of optimizing target allocation is to make each AUV save itself as much as possible, avoid repeated attacks, and achieve maximum damage to the whole targets. Since antagonism is complex, many factors must be considered in the process, such as damage probability, total path cost, total time, total energy consumption, and so on. Minimizing the total path cost requires completing the global task with minimal path cost. Reducing the whole time to complete a global task is to expect to complete all tasks in the shortest amount of time. When AUVs confront, minimization of the total energy consumption of the weapon is the least requirement. Therefore, target allocation is a multi-constrained multivariate combinatorial optimization problem. Constructing a multi-objective optimization function and using a multi-objective optimization method can provide multiple allocation strategies for commanders. Moreover, it can adapt to the battlefield situation better.

In the past, the weighted method was usually used to solve the multiple targets allocation, which transformed multi-objective optimization into a single objective optimization problem. Although the strategic solution obtained by this method often can achieve a high damage probability, it also may cause mutual loss. In war confrontation, the aim is assumed to minimum own side loss and maximize the opponent damage probability. Therefore, factors that should be considered include maximum profit, minimum loss, and so on. As multiple objective functions should be considered at the same time, it is appropriate to solve the problem using the multi-objective optimization theory.

Effective communication is the basis and guarantee for achieving multi-AUV collaborative confrontation. Due to the complex underwater environment and the AUV movement, communication between AUVs is limited in most cases. It is necessary to discuss the influence of communication constraints on the synergy of AUV. In the underwater confrontation, the threat target state parameter obtained by the attacker AUV is provided by the sonar sensors. Due to the underwater communication delay, the attacker gets the threat target position $x(t_{0}+\Delta t)$, and the actual target position is $x(t_{0})$, Δt is the delay time of underwater communication. The communication delay influence factor $D=\frac{\sqrt{{\left(x_{s}-x_{0}\right)}^{2}+{\left(y_{s}-y_{0}\right)}^{2}+{\left(z_{s}-z_{0}\right)}^{2}}}{\sqrt{x_{0}^{2}+y_{0}^{2}+z_{0}^{2}}}$ is introduced to evaluate the influence of underwater communication delay on the underwater AUV confrontation effect. Where $\left(x_{s},y_{s},z_{s}\right)$ represents the target position of the sonar sensor for attacking the AUV and $\left(x_{0},y_{0},z_{0}\right)$ represents the actual position of the target. The mathematical description of multi-objective decision-making model is shown as follows:

Suppose that in a naval battle, multiple AUVs of R side cooperate to attack multiple targets of B side, the AUV formation is composed of $N_{m}$ AUVs. The total number of weapons is m and the number of threat target is $N_{t}$. In one confrontation, the minimum opponent residual threat probability and the minimum number of weapons consumed by AUVs are selected as objective functions to construct the multi-objective decision model:

(13) $$\left\{ \begin{array}{l} \min F(\pi )=(P(\pi ),W(\pi ),D(\pi ))\\ P(\pi )=\min\frac{1}{N_{t}}\sum_{j=1}^{N_{t}}\left[\prod_{l=1}^{m}{\left(1-(1-D(\pi ))P_{ij}\right)}^{\delta (i-j)}\right]\\ W(\pi )=\min\sum_{i=1}^{N_{m}}\sum_{l=1}^{m}\delta (i-j)\\ D(\pi )=\frac{\sqrt{{\left(x_{s}-x_{0}\right)}^{2}+{\left(y_{s}-y_{0}\right)}^{2}+{\left(z_{s}-z_{0}\right)}^{2}}}{\sqrt{x_{0}^{2}+y_{0}^{2}+z_{0}^{2}}} \end{array}\right.\;s.t.\prod_{l=1}^{m}{\left(1-P_{ij}\right)}^{\delta (i-j)}\leq K_{P}$$

P(π) is the minimum opponent residual threat probability, $P_{ij}$ is the damage probability of $i^{th}$ AUV attacking $j^{th}$ target. W(π) is the minimum number of weapon consumed. $K_{P}$ is the opponent residual threat threshold. The term δ(i−j) is the Kronecker delta defined by

(14) $$\delta (i-j)=\{\begin{array}{ccc} 0 & if & i\neq j\\ 1 & if & i=j \end{array}$$

It is used to indicate that unit *i* of the *R* side has been assigned to target unit *j* of the *B* side. Generally, performance indicators P(π) and W(π) are contradictory. Minimizing survival probability means that it will consume more weapons, while minimizing consumed weapons may indicate that the survival antagonistic probability increases. Therefore, it does not exit the unique solution to optimize the two performance indices at the same time. The optimal solution is a Pareto solution set that may contain more than one element.

## 3. MODPSO for Solving Underwater Cooperative Target Allocation

The research on decision-making for underwater cooperative confrontation in naval warfare aims to find the most suitable target allocation strategies according to the changing battlefield situation and the decision preferences of the commander. It is a non-continuous discrete problem. The research on target allocation with PSO mainly focuses on the continuous domain, which is to say, the variables that describe the particle state and the characteristics of motion are continuous. There is little research on discrete decision-making. In this paper, the multi-objective discrete particle swarm optimization algorithm is used to solve the underwater multi-objective decision-making model.

The velocity and position update formula of the fundamental particle swarm optimization algorithm is difficult to express the discrete domain problem such as coordinated multi-objective allocation. Therefore, this paper draws on the idea of a genetic algorithm and designs the particle position and velocity update formula that accords with the discrete domain characteristics of the problem. At the same time, this paper also combines the discrete particle swarm optimization algorithm with the multi-objective optimization algorithm to solve the underwater cooperative confrontation decision-making problem. The particle coding, update of the speed, position, individual particle leader, and global particle leader selection are detailed in the following.

### 3.1. Particle Coding

The decision variables must be coded to clearly express the physical meaning of particles. Particle coding includes the position and velocity of the particle. Each particle represents a possible solution. How to make particles correspond to feasible solutions is the key to solving problems. During underwater antagonism, each weapon is assigned once, and each target is attacked by at least one weapon. The AUV that performs the task corresponds with the threat target. The nature number coding form is used in this paper.

Nature number coding is applied to illustrate the AUV number assigned to the threat targets. The particle position represents a candidate scheme for target allocation, in other words, which threat target is allocated to which AUV. The length of each particle is equal to the total number of targets.

Assume that the total number of particles is R, the *r*^th^ particle position vector is $X_{r}=[\begin{array}{cccccc} X_{r1} & X_{r2} & \ldots & X_{ri} & \ldots & X_{rN_{t}} \end{array}]$, $X_{ri}(i=1,2,\ldots ,N_{t})$ is an integer between 0 and $N_{t}$.

**Figure 2:**
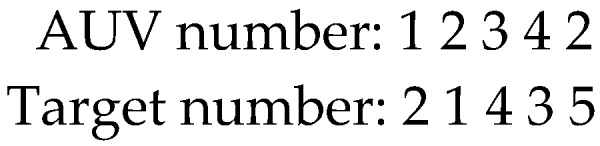
Task allocation instance.

For example, there are four AUVs and five threat targets. Figure 2 shows possible particle coding is $X_{r}=[\begin{array}{ccccc} 1 & 2 & 3 & 4 & 2 \end{array}]$. It represents the first AUV attacks the second target, and the second AUV attacks the first target, etc.

### 3.2. Particle Update Formula

Each particle in the PSO may adjust its position according to its own and neighboring-particles experience. Then it moves toward its best position or the best position of its neighbor. According to the characteristics of underwater cooperative confrontation, combining with the genetic algorithm, the position and the velocity update formulas of the particle are redefined. The velocity of the particle is defined as the changing rate of the position of the particle.

(15) $$V_{i}^{k+1}(t)=c_{2}\to F_{3}(c_{1}\to F_{2}((\omega \to F_{1}(X_{i}^{k}(t))),p_{i}(t)),p_{g}(t))$$

(16) $$X_{i}^{k+1}(t)=X_{i}^{k}(t)+V_{i}^{k+1}$$

$\omega \to F_{1}(X_{i}^{k}(t))$ is inertia part, ω indicates that the velocity of the particle is a replacement operation with probability ω. Define Φ(t) as intermediate variables, and $rand_{1}()$ is a random number in [0,1]. If $rand_{1}()<\omega$, then $\Phi (t)=F_{1}(X_{i}^{k}(t))$, else $\Phi (t)=X_{i}^{k}(t)$. That is

(17) $$\Phi (t)=\{\begin{array}{ll} F_{1}(X_{i}^{k}(t)) & rand_{1}(\;)<\omega \\ X_{i}^{k}(t) & rand_{1}(\;)\geq \omega \end{array}$$

$c_{1}\to F_{2}((\omega \to F_{1}(X_{i}^{k}(t))),p_{i}^{k}(t)$ is self-awareness part. The particle adjusts its position according to the individual extreme value $p_{i}(t)$. $c_{1}\to F_{2}((\omega \to F_{1}(X_{i}^{k}(t))),p_{i}(t)$ indicates that the velocity of the particle is a cross operation with probability $c_{1}$. Define Ψ(t) as intermediate variables, and $rand_{2}()$ is a random number in [0,1]. If $rand_{2}()<c_{1}$, then $\Psi (t)=F_{2}(\Phi (t),p_{i}(t))$, else Ψ(t)=Φ(t). That is

(18) $$\Psi (t)=\{\begin{array}{ll} F_{2}(\Phi (t),p_{i}(t)) & rand_{2}(\;)<c_{1}\\ \Phi (t) & rand_{2}(\;)\geq c_{1} \end{array}$$

$c_{2}\to F_{3}(c_{1}\to F_{2}((\omega \to F_{1}(X_{i}^{k}(t))),p_{i}(t)),p_{g}(t))$ is social awareness part. The particle adjusts its position according to the global optimal extremum $p_{i}(t)$. $c_{2}\to F_{3}(c_{1}\to F_{2}((\omega \to F_{1}(X_{i}^{k}(t))),p_{i}(t)),p_{g}(t))$ indicates that the velocity of the particle is a cross operation with probability $c_{2}$. Define $rand_{3}()$ is a random number in [0,1]. If $rand_{3}()<c_{2}$, then $V_{i}^{k+1}(t)=F_{3}(\Psi (t),p_{g}(t))$, else $V_{i}^{k+1}(t)=\Psi (t)$. That is

(19) $$V_{i}^{k+1}(t)=\left\{ \begin{array}{ll} F_{3}(\Psi (t),p_{g}(t)) & rand_{3}(\;)<c_{2}\\ \Psi (t) & rand_{3}(\;)\geq c_{2} \end{array}\right.$$

In the iterative process, $p_{i}(t)$ and $p_{g}(t)$ are continuously updated, and the final output $p_{g}(t)$ is the global optimal solution.

### 3.3. Individual Particle Leader Renewal

The individual particle leader is the best particle position from the initial to the present iteration times. It can be regarded as the memory of the particle. The individual particle leader is renewed based on the constrained dominance relationship. Suppose $p_{i}$ is the individual particle leader of $X_{i}^{k}$, the k+1 generation of new particles is $X_{i}^{k+1}$. The individual particle leader $p_{i}^{k+1}$ is replaced by $X_{i}^{k+1}$ when $p_{i}$ is constrained dominance by $X_{i}^{k+1}$; $p_{i}^{k+1}$ is replaced by $X_{i}^{k+1}$ or $p_{i}$ randomly, when neither of them is dominant from each other, else $p_{i}^{k+1}$ is replaced by $p_{i}$.

### 3.4. Reserve Solution Set Renewal

Since the feasible reserve set applies an optimum solution, the feasible reserve set is updated by using the Pareto dominance relationship in the final process of the algorithm calculation. Firstly, combine the existing elements in the feasible reserve set and the new feasible solutions in particle swarm into a new population. Secondly, the non-dominant elements in the population are selected, and these elements are preserved in the feasible reserve set by using the Pareto dominance relationship. If the number of items in the feasible reserve set exceeds its inherent capacity $N_{a}$, the method in [33] is used to calculate the crowding distance of each element. Keep the most sparsely distributed elements, that is, the elements with excessive congest distance values will be kept.

The infeasible reserve set is updated based on the updated feasible reserve set. Combine the existing elements in the infeasible reserve set and the new infeasible solutions in the particle swarm into a new population, and reselect the elements from the population to carry them out into the infeasible reserve set. The non-feasible solution which dominates the elements of the feasible reserve set, as well as the non-feasible solutions which are not dominated by the feasible reserve set elements and are located in the sparse area, are preserved in the non-feasible reserve set.

### 3.5. Global Particle Leader Selection

In the process of optimization, if the element in the infeasible reserve set is chosen as a global particle leader, the global development ability of the algorithm will be enhanced. If the element of the feasible reserve set is selected as the global particle leader, it can guide the particle to develop the feasible region deeply, and improve the quality of the existing feasible noninferior solution.

This paper adopts a dynamic allocation strategy based on the selection probability, for balancing the above two selection approaches effectively. In the iteration of the algorithm, the global leader of the particle is selected from the infeasible reserve set and the feasible reserve set with probability $p_{st}(0\leq p_{st}\leq 1)$ and $1-p_{st}$ respectively.

(20) $$p_{st}=p_{st1}-p_{st2}\frac{k}{k_{\max}}$$

where $k_{\max}$ is the algorithm termination iterations, $p_{st1}$ and $p_{st2}$ are constant numbers satisfying $0\leq p_{st1}\leq p_{st2}\leq 1$. In the beginning, the global particle leader is selected from the infeasible reserve set with a high probability, which will help to maintain the diversity of particles and enable the particle to search for more feasible regions, including isolated feasible regions. As the number of iterations increase, the algorithm gradually focuses on the feasible reserve set. It means that the algorithm will have more chances to search for the feasible region in the late iteration, to deeply explore the existing feasible non-inferior solutions. Experiments show that the algorithm has good performance when $p_{st1}$ and $p_{st2}$ are 0.7 and 0.6, respectively. When the non-feasible reserve set is empty, their global leader will be selected from the feasible reserve set for all particles. Similarly, when the feasible reserve set is empty, the global leader will be chosen from the infeasible reserve set for all particles.

**Figure 3:**
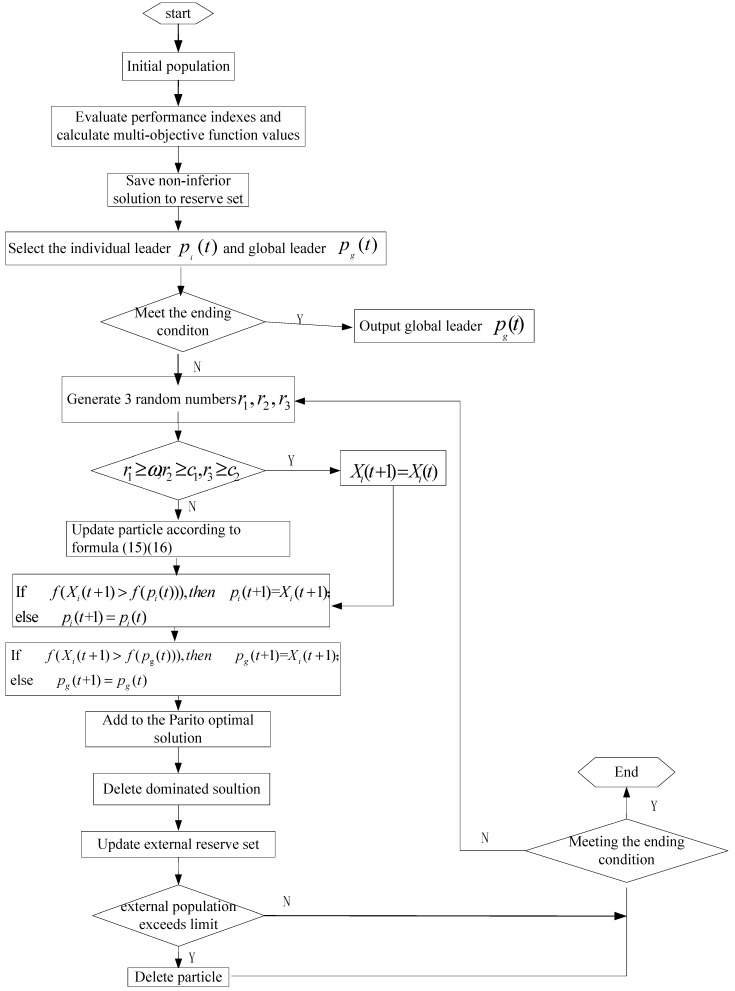
Chart of the multi-objective discrete particle swarm optimization (MODPSO) algorithm.

The proposed multi-objective discrete particle swarm optimization algorithm is shown in Figure 3.

This paper combines the discrete particle swarm optimization (DPSO) algorithm with the multi-objective optimization algorithm to solve the confrontation decision-making problem under the influence of underwater communication delay. They are well adapted to the characteristics of decision-making discretization and take into account the decision preferences of the commander.

## 4. Simulation Experiments

To illustrate the correctness of the underwater cooperative confrontation model established and the effectiveness of the MODPSO algorithm, a typical scenario simulation is implemented.

### 4.1. Simulation Setup

It postulates that we have five AUVs armed with 15 weapons, and find ten targets by sensors. The target residual threat threshold is 0.1. Table 1 shows the weapon number of AUV formation. Table 2 shows the damage probability of each weapon.

This article provides a comparison between the MODPSO algorithm and the NSGA-II algorithm to demonstrate the effectiveness and efficiency of the proposed algorithm. In the NSGA-II, the population size is 100, the evolutionary population is the same as the external population, and the number of iterations is 100. The crossover probability is 0.82, and the probability of variation is 0.15. In the MODPSO, the population size is 100, the number of iterations is 100, and each particle represents an underwater cooperative confrontation strategy. The threshold of the MODPSO external population is 25. In the simulation, the confrontation strategy exceeding the damage probability threshold is deleted. Choose from unallocated weapons to hit targets that do not reach the probability of damage. We discuss the impact of communication delay factors on underwater cooperative countermeasure strategies in different ranges.

### 4.2. Simulation Results

**Figure 4:**
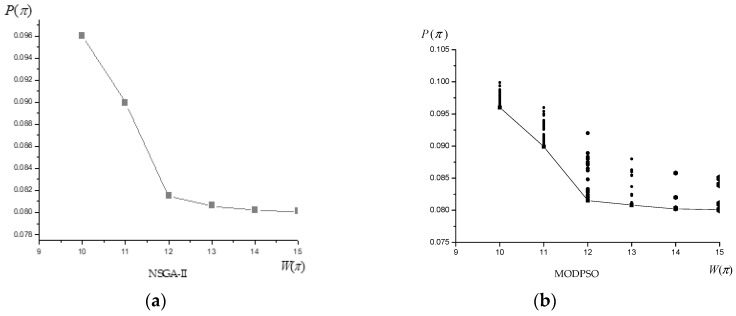
Results of the two algorithms.

Figure 4 shows the simulation results. The abscissa is the objective function W(π), and the ordinate is the objective function P(π). The NSGA-II and MODPSO converge on the found Pareto optimal solution set, respectively, and shown by the connection. The evolutionary population of the MODPSO is shown in the point set of the figure. We can see that the MODPSO algorithm has better searching ability and particle diversity.

The MODPSO algorithm is used to solve the issue under different group sizes and iterations, which runs 50 times, respectively. Table 3 shows the average running time of the algorithm.

**Figure 5:**
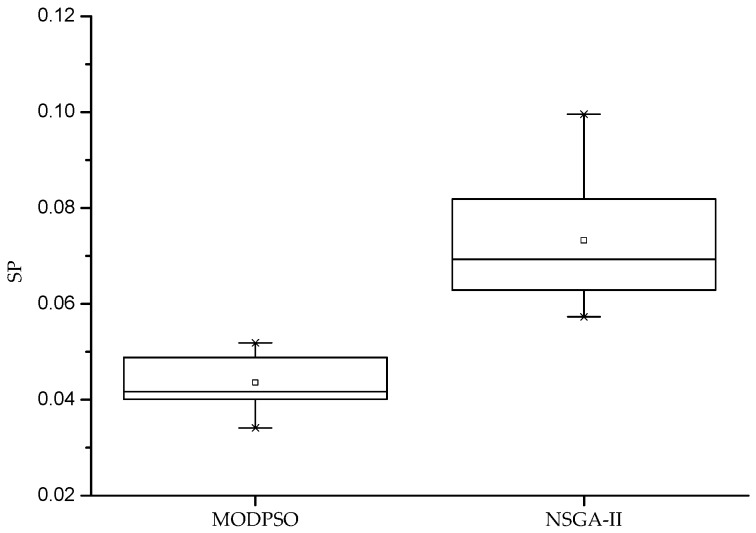
Distribution uniformity of Pareto optimal set of algorithms.

As can be seen from Table 3, the MODPSO algorithm can effectively meet the real-time requirements of the underwater cooperative confrontation decision-making model. The distribution breadth index SP is used to evaluate the distribution uniformity of the Pareto solution. The smaller the SP value, the more uniform the Pareto solution distribution. Under the condition that the population size is 100 and the number of iterations is 100, the algorithm runs 50 times, independently. The statistical results of SP values are as follows:

As can be seen from Figure 5, the distribution of the Pareto optimal solution set obtained by the MODPSO algorithm for solving the underwater cooperative confrontation decision is the most uniform and stable.

### 4.3. Analysis and Discussion

**Figure 6:**
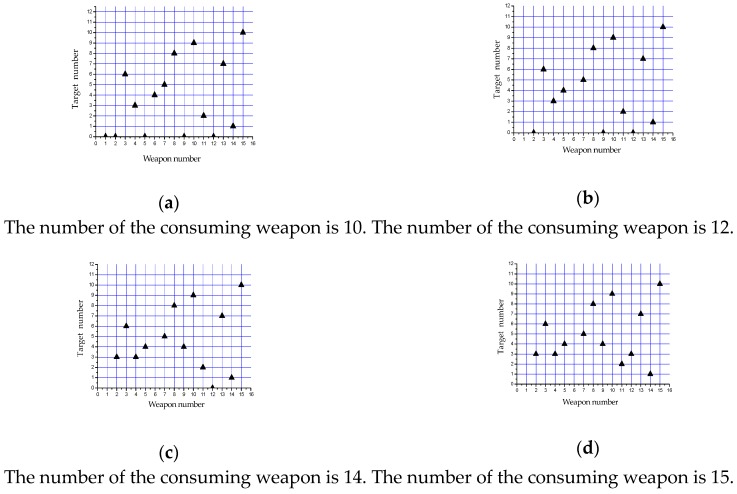
Target allocation scheme under different weapon consumption.

In the process of confrontation, there is no need to assign targets to all weapons. The more weapons you consume, the lower the survival probability is. When the expected damage effect is achieved, it is not necessary to waste all the weapons. Therefore, the firepower is preserved. It saves strength for attacking subsequent targets. Depending on the expected opponent residual threat threshold index, the established multi-objective decision-making model can save power sources. Figure 6 shows the optimization strategies under different weapon consumption. The symbol “▲” indicates that the weapon is assigned to the corresponding target. All of them are feasible, and it can be selected by the commander depending on the specific situation.

**Figure 7:**
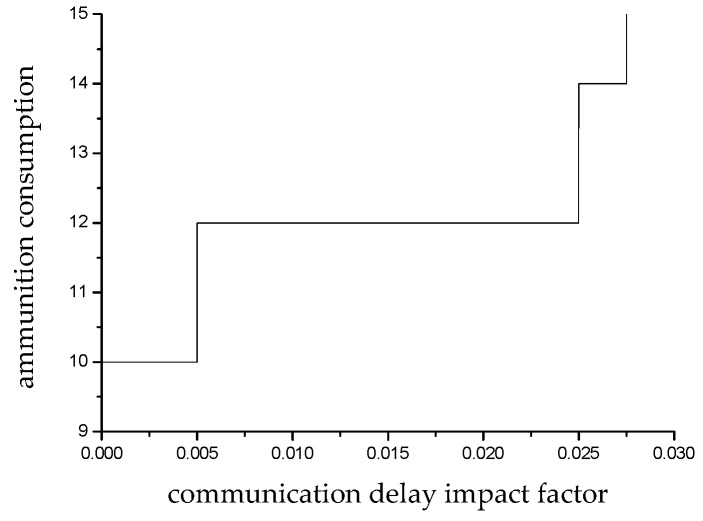
Weapon consumption under different communication delay influence factors.

Figure 7 shows the impact of the communication delay impact factor on the choice of underwater countermeasures. It can be noted that when the communication delay influence factor is lower than 0.005, the strategy choice is approximately equal to the ideal state, and the ammunition consumption amount is 10 to achieve the operational expectation. When the communication delay impact factor is between 0.005 and 0.0245, the amount of ammunition consumption needs to be increased to 12. When the communication delay impact factor is between 0.0245 and 0.0274, the amount of ammunition consumption needs to be increased to 14. When the communication delay impact factor is 0.0275, all 15 munitions carried need to be launched, and at this time, the target residual threat probability is just at the critical minimum threat threshold. If the communication delay impact factor is higher than 0.0275, the ammunition carried can no longer complete the expected damage effect on the targets.

The underwater cooperative countermeasure strategy can be selected by the multi-objective discrete particle swarm optimization algorithm. Due to the complexity of underwater cooperative confrontation, it is necessary to consider the influence of communication delay on the choice of underwater countermeasures. The commander can choose the optimal confrontation strategies based on the battlefield situation, which is more in keeping with the actual naval warfare situation.

## 5. Conclusions

This paper studies the decision-making problem of underwater cooperative confrontation deeply. Moreover, it establishes an underwater multi-objective collaborative confrontation decision model with minimum opponent residual threat probability and minimum weapon consumption as the objective functions, which is constrained by communication delay. According to the discontinuity of the confrontation strategy, the particle coding form is improved, and the discrete particle swarm optimization algorithm is used to find the optimal solution. This paper analyzes the choice of strategies that are influenced by communication delay factors in different value ranges. Under the premise of satisfying the residual threat threshold of the opponent, due to the communication delay, the weapon consumption will be increased even when the other conditions are the same. The simulation results show that underwater communication delay has an inevitable impact on the choice of underwater countermeasures strategies. The established multi-objective decision model helps us make priority decisions based on actual combat, reduce weapons consumption, and save resources while meeting the expectations of target damage. This research has certain practical significance and provides a more reasonable research idea for effectively solving the problem of underwater coordinated confrontation decision-making.

## Figures and Tables

**Table 1:** Number of autonomous underwater vehicle (AUV) formation armed with weapons.

AUV Formation	Weapons Number
A_1_	W_1_~W_3_
A_2_	W_4_~W_6_
A_3_	W_7_~W_9_
A_4_	W_10_~W_12_
A_5_	W_13_~W_15_

**Table 2:** Probability of each weapon.

	Target	1	2	3	4	5	6	7	8	9	10
Weapon											
1		0.53	0.82	0.91	0.85	0.75	0.62	0.84	0.82	0.78	0.64
2		0.76	0.81	0.91	0.75	0.91	0.78	0.80	0.64	0.60	0.83
3		0.83	0.74	0.86	0.53	0.84	0.93	0.60	0.81	0.74	0.80
4		0.83	0.81	0.92	0.84	0.86	0.83	0.60	0.78	0.65	0.67
5		0.71	0.71	0.72	0.90	0.78	0.66	0.86	0.69	0.84	0.82
6		0.82	0.60	0.56	0.92	0.57	0.73	0.62	0.87	0.75	0.64
7		0.85	0.83	0.60	0.78	0.87	0.84	0.79	0.65	0.60	0.78
8		0.81	0.72	0.62	0.91	0.88	0.67	0.78	0.90	0.84	0.58
9		0.65	0.63	0.84	0.87	0.57	0.72	0.64	0.87	0.82	0.57
10		0.83	0.84	0.88	0.80	0.73	0.72	0.87	0.78	0.91	0.67
11		0.85	0.88	0.78	0.86	0.58	0.79	0.81	0.80	0.82	0.64
12		0.84	0.87	0.89	0.67	0.84	0.89	0.56	0.75	0.64	0.85
13		0.62	0.71	0.84	0.57	0.78	0.87	0.88	0.72	0.65	0.62
14		0.93	0.85	0.79	0.67	0.83	0.81	0.64	0.85	0.84	0.86
15		0.57	0.71	0.62	0.87	0.58	0.79	0.86	0.84	0.72	0.90

**Table 3:** Mean running time.

Number of Iterations	Population Size		
	50	100	200
50	0.55634	1.2211	2.7173
100	1.2538	2.6963	5.2719
200	2.5843	4.8592	9.5971
